# Plant-Based Natural Product Chemistry for Integrated Pest Management of *Drosophila suzukii*

**DOI:** 10.1007/s10886-019-01085-1

**Published:** 2019-07-01

**Authors:** Ian W. Keesey, Nanji Jiang, Jerrit Weißflog, Robert Winz, Aleš Svatoš, Chen-Zhu Wang, Bill S. Hansson, Markus Knaden

**Affiliations:** 1 Department of Evolutionary Neuroethology, Max Planck Institute for Chemical Ecology, Hans-Knöll-Straße 8, D-07745 Jena, Germany; 2 State Key Laboratory of Integrated Management of Pest Insects and Rodents, Institute of Zoology, Chinese Academy of Sciences, 1 Beichen West Road, Chaoyang District, Beijing, 100101 China; 3 Mass Spectrometry/Proteomics Research Group, Max Planck Institute for Chemical Ecology, Hans-Knöll-Straße 8, D-07745 Jena, Germany; 4 The New Zealand Institute for Plant & Food Research, 120 Mt. Albert Road, Mt. Albert, Auckland 1025, Private Bag 92169, Auckland, 1142 New Zealand

**Keywords:** Olfaction, Chemical ecology, Spotted wing *Drosophila*, Insect behavior, Parasitoid, Oviposition, IPM

## Abstract

Since the first reports of damage by *Drosophila suzukii*, the spotted-wing *Drosophila* (SWD), over a decade ago in Europe, widespread efforts have been made to understand both the ecology and the evolution of this insect pest, especially due to its phylogenetic proximity to one of the original model organisms, *D. melanogaster*. In addition, researchers have sought to find economically viable solutions for the monitoring and management of this agricultural pest, which has now swept across much of Europe, North America and Asia. In a new direction of study, we present an investigation of plant-based chemistry, where we search for natural compounds that are structurally similar to known olfactory cues from parasitoid wasps that in turn are well-described ovipositional avoidance cues for many *Drosophila* species. Here we test 11 plant species across two plant genera, *Nepeta* and *Actinidia*, and while we find iridoid compounds in both, only those odorants from *Actinidia* are noted to be detected by the insect antenna, and in addition, found to be behaviorally active. Moreover, the *Actinidia* extracts resulted in oviposition avoidance when they were added to fruit samples in the laboratory. Thus we propose the possible efficacy of these plants or their extracted chemistry as a novel means for establishing a cost-effective integrated pest management strategy towards the control of this pest fly.

**Electronic supplementary material:**

The online version of this article (10.1007/s10886-019-01085-1) contains supplementary material, which is available to authorized users.

## Introduction

Since its identification in Spain and Italy in 2008 (Cini et al. 2012), *Drosophila suzukii*, the spotted wing *Drosophila* (SWD), has continued to spread and remain a consistent problem throughout Europe for agricultural and commercial businesses that produce a wide variety of berry fruits or their associated products. Eleven years later, as of 2019, this pest insect has been unrelenting in its invasion and its spread of economic damage within not just Europe (Tait et al. 2018), but also across North America and Asia (Cloonan et al. 2018). Since then, a variety of publications have increased our knowledge regarding its chemical ecology (Adrion et al. 2014; Crava et al. 2016; Hwang et al. 2005; Karageorgi et al. 2017; Keesey et al. 2015; Ramasamy et al. 2016). However, even given the prevalence of *D. suzukii* as a target for research during the last decade, there is still a large gap in applicable technology for monitoring and control of this insect pest.

Commercial synthetic production of compounds known to be repellent towards the *Drosophila* genus, such as geosmin (Stensmyr et al. 2012), are prohibitively expensive to produce for large-scale usage in the field (Cloonan et al. 2018; Wallingford et al. 2016a, b), and their application on the fruit shortly before harvest (*i.e.* when *D. suzukii* mainly infests the berries) might have a negative impact on fruit quality (Diepenbrock et al. 2017; Leach et al. 2018). Forinsect pests, natural-product chemistry, including both microbial-derived and plant-derived sources have been shown to be efficient (Abdel-Sattar et al. 2010; Maia and Moore 2011; Pavela 2016). For example, the leaves of pepper trees and their extracts have been used to repel house flies (*Musca domestica*) in Ethiopia (Abdel-Sattar et al. 2010), and peppermint oils have been shown to be repellent towards *D. suzukii* in a laboratory setting (Renkema et al. 2016). However additional, economically viable strategies to combat *D. suzukii* still need to be identified, as most research has focused on attractive odors for monitoring or bait and kill strategies for population control (Klick et al. 2019; Landolt et al. 2012;).

An area of rapidly growing research has been the study of biocontrol options for *D. suzukii* using either existing, native parasitoid wasps (*e.g.* those found in Europe) or other parasitoids from evolutionary associations within the original habitats occupied by *D. suzukii* (*e.g.* those parasitoids from China, Korea and Japan). Here, although numerous parasitoid wasp species have been examined, there appear to be barriers to the effectiveness of these biocontrol agents (Asplen et al. 2015; Daane et al. 2016), especially due to variation in the immune system of *D. suzukii* and its superior ability to encapsulate wasp eggs relative to *D. melanogaster* adults (Chabert et al. 2012; Kacsoh and Schlenke 2012). However, there may still be future success in this tactic, once a suitable species of parasitoid wasp has been uncovered to more effectively manage these flies in natural and agricultural ecosystems. In association with parasitoids, a study that recently focused on *D. melanogaster* identified three pheromone components of the *Drosophila*-specific parasitoid, *Leptopilina boulardi* (Hymenoptera: Figitidae: Eucoilinae), where each of the three pheromone components (*i.e.* iridomyrmecin, nepetalactol, and actinidine) from the wasp body wash was shown to activate the same parasitoid-specific olfactory sensory neuron (OSN) in *D. melanogaster*. Moreover, it was shown that this neural activation pathway in turn leads to oviposition avoidance of these parasitoid odorants by the adult fly (Ebrahim et al. 2015). Importantly, this study also examined behaviors related to other flies within the genus, including *D. suzukii*, where the presence of these three wasp pheromones also generated significant avoidance, both in larvae and adults. Interestingly, although being shown to act as sex pheromone in the wasp, for at least some parasitoid species within the *Leptopilina* genus, the same compounds seem to also be used as marking pheromones that help to avoid competition for Drosophilid hosts (Pfeiffer et al. 2018). However, again, the commercial synthetic production of these known repellent wasp compounds is both expensive and labor-intensive, as would be the collection of these odors from the mass production of parasitoid wasps, which themselves are much smaller than a single *D. melanogaster* adult (Ebrahim et al. 2015; Stökl et al. 2012;). Therefore, collection of these odorants from these tiny Hymenopterans may prove to be too inefficient for large-scale production.

As iridoid compounds that bear strikingly similar structural chemistry towards the wasp pheromones have also been described in plants like catnip, *e.g. Nepeta* Lichman et al. 2018; Sherden et al. 2018; Shim et al. 2000;), and kiwifruit, *e.g. Actinidia* (Lu et al. 2007; Matich et al. 2003; Tatsuka et al. 1990; Twidle et al. 2017), we therefore hypothesized that these plant genera may provide a natural source of functional odorants to repel *D. suzukii* adults. Commercial products already exist from *Nepeta* plants, such as dried *Nepeta* leaves, as well as extracted oils or synthetic compounds that have similar bioactivity to those identified from *Nepeta* varieties, where all products are sold as cat toys or feline attractants (Bol et al. 2017). Catnip oil extracts have also been shown previously to possess some behavioral repellency against other Dipterans, such as the stable fly, *Stomoxys calcitrans*, and other biting flies, which are pests of livestock (Zhu 2011). However, to our knowledge, no integrated pest management (IPM) strategy has ever been examined utilizing kiwifruit plant materials.

In order to test our hypotheses that natural, plant-based chemistry may provide possible IPM solutions towards *D. suzukii*, we therefore searched for parasitoid-like iridoid compounds in the plant extracts from seven total *Nepeta* plant species as well as from four total *Actinidia* species. In addition, we tested whether any of these plant-produced odorants could activate the parasitoid-specific olfactory sensory neuron pathway in the adult fly. Lastly, we sought to examine whether these plant-based odorants were sufficient to induce ovipositional avoidance by *D. suzukii* adults in the laboratory.

## Methods and Materials

### Plant Sample Extraction

Fresh samples of 8 varieties across 7 species within the *Nepeta* genus were collected from potted plants grown outdoors, including leaves as well as inflorescences, which were each collected separately. Plant extracts were generated using 1 hr washes of cut plant material (2–3 g) in hexane or separately via washes in methanol, and collections were aided by periodic mixing (vortex genie 2; www.scientificindustries.com). Extraction protocols for plant materials followed established methods and solvents for iridoid compounds as well as from the literature related to these plant genera (Hallahan et al. 1998; Lu et al. 2007; Tatsuka et al. 1990;). Liquid extractions were filtered to remove any loose particulates, and then stored at −20 °C until ready for GC-MS and insect trials. Fresh samples of flowers and leaves from 4 plant species and multiple genotypes within the *Actinidia* genus were also collected and then shipped from New Zealand to Germany during the northern hemisphere summer 2018 (winter in New Zealand). The samples were collected via the Plant & Food Research team in Auckland. These samples were kept frozen in temperature monitored containers during transport (www.WorldCourier.com), with alarms set to trigger if temperatures reached above −15 °C, which occurred only once (just following arrival in Germany). Frozen *Actinidia* samples were then immediately stored again at −80 °C until ready for processing in the laboratory. Tissue samples were broken off from frozen *Actinidia* materials (leaves and flowers; 2–3 g) and plant extracts were collected using 1 hr washes in hexane and methanol solvent as previously described for *Nepeta* plant tissues.

### Gas Chromatography Mass Spectrometry (GC-MS)

Chemical analyses were performed on all plant extract collections as described previously (Keesey et al. 2015, 2017; Qiao et al. 2019), where plant materials were generated via external washes (as opposed to plant materials being ground, crushed or homogenized). The NIST mass-spectral library identifications were confirmed with chemical standards where possible, including the high-purity synthetic isomers of the parasitoid pheromones from *Leptopilina boulardi* wasps, which were previously identified and published from our Institute (Ebrahim et al. 2015). Plant extracts for all *Actinidia* and *Nepeta* species and tissue types were prepared in both hexane and methanol solvents (to provide additional diversity across polarity, as well as to maximize chemical identification and differentiation of each compound within these plant samples). Moreover, all plant samples were run across both HP5 and HP-INNOWax GC columns during our chemical analyses. Raw data files (Agilent ChemStation) are available with the online version of this publication. Chemical standards were generated from in-house laboratory sources (Mass Spectrometry Research Group, Max Planck Institute for Chemical Ecology) and were of the highest purity possible. In total 6 chemical standards were produced, including: (4*R*,4a*R*,7*R*,7a*S*)-(−)-iridomyrmecin (87% pure), a 1:1 mix of (4*S*,4a*R*,7*R*,7a*S*)-(+)-isoiridomyrmecin and (4*R*,4a*R*,7*R*,7a*S*)-(−)-iridomyrmecin, (*R*)-actinidine (95% pure), (*R*)-actinidine (20% EtOAc) and (1*S*,4a*R*,7*R*,7a*S*)-nepetalactol (Supplementary Figure 2). Syntheses were conducted as described previously for stereoisomers of iridomyrmecin (Stökl et al. 2012; Fischman et al. 2013) as well as for actinidine and nepetalactol (Beckett et al. 2010) using (*R*)-citronellal as starting material. Dilutions of each of the synthetic odors were prepared in hexane for both electrophysiological and behavioral trials, and synthetic chemicals were stored with nitrogen in sealed containers and kept in the dark at -80 °C when not in use.

### Fly Stocks

Wildtype *D. suzukii* (14023–0311.01) were obtained from the former University of California San Diego Drosophila Stock Center, which is now the National Drosophila Species Stock Center (Cornell University; http://blogs.cornell.edu/drosophila/). All experiments with wildtype *D. melanogaster* were carried out with the Hansson Canton-S (CS) laboratory strain, which were originally obtained in 2008 from the Bloomington Drosophila Stock Center (www.flystocks.bio. indiana.edu). Fly stocks were maintained according to previous studies (Keesey et al. 2015, 2019), and for all behavioral experiments we used 4 to 7 day-old flies.

### Single-Sensillum Recordings (SSR)

In order to assess olfactory similarities and differences between these two *Drosophilia* species, we conducted single-sensillum recordings of both flies. Adults were held immobile within plastic pipette tips, with only the head of the fly exposed, where the third antennal segment (funiculus) and compound eye were both stabilized against a glass cover slip to aid tungsten electrode penetration. Sensillum type was identified using established diagnostic odors and via searching established antennal regions of highest density probability (Ebrahim et al. 2015; Lin and Potter 2015). A reference electrode (tungsten) was inserted into the compound eye closest to the targeted antenna, while a recording electrode (tungsten) was used to pierce individual sensillum types along the antenna for potential screening and identification using the appropriate diagnostic odors. Odor stimulus preparation and delivery for SSR experiments followed previously established procedures (Keesey et al. 2017).

### Oviposition Assays

As a means to examine the role these iridoids play in the ecology of *D. suzukii*, we sought to test egg-laying decisions in the presence of these odors. Experiments were carried out in large mesh cages (50 cm × 50 cm × 50 cm; https://shop.bugdorm.com/bugdorm-4f4545-insect-rearing-cage-p-31.html, BugDorm-44,545 F) which were placed inside walk-in growth chambers (12 hr Light:Dark, 70% humidity, 23 °C). Oviposition choice plates were generated with standard *Drosophila* diet, with a single freshly smashed blueberry in the center, and each blueberry had either 50 μl solvent control (hexane) or 50 μl of treatment (also in hexane) added over it (similar to previous oviposition methods described for *D. suzukii* experiments (Karageorgi et al. 2017)). However, we had several difficulties initially with establishing oviposition with *D. suzukii*, thus we made several modifications. First, each cage had moistened white tissue paper in the center. This reduced desiccation pressures over the behavioral trial by providing a constant source of water, it provided a surface texture that reduced turtling of flies (*e.g.* stuck on their backs), as opposed to the slippery plastic bottom surface of the cages, and the tissue paper also generated a reproducible distance between control and treatment plates (20 cm). This strategy boosted survivorship of adult flies to over 90% during these behavioral trials. Second, our packaged blueberries varied considerably in size and color, thus control and treatment blueberries were selected to match each other in size, weight and color. Lastly, despite our best efforts to utilize agar plates, or transparent media to aid in egg counting, we could not get sufficient oviposition from *D. suzukii* females using these media types. Therefore, we chose to use the same standard diet that the flies are already reared upon, which we first transferred to oviposition plates and spread evenly, then provided texture, and finally placed our freshly smashed blueberry in the center (Fig. 5d). Each cage had a total of 20 females and 10 males released inside to ensure optimal mating status (where flies were selected after 2–4 min of anesthesia by cooling at −20 °C; no CO_2_ was used to prepare the flies for oviposition trials). Appropriate age and reproductively viable females were selected as those which released and displayed a single egg from their ovipositor during anesthesia. After transferring adults to the cages, flies were given 48 hr to adjust to the enclosures as well as to lay eggs, and then both the control and treatment plates were removed for counting and subsequent photo analyses. Eggs that hatched prior to plate removal were still countable, as the chorion (shell) was still visible after larval emergence, and although most intact eggs were deeply buried by the females in our substrate, the pairs of breathing tubes were still visible to facilitate accurate egg counts. We also found it useful to use a black and white camera, as opposed to full color, in order to better discern eggs that had been completely buried (Fig. 5d, e). The oviposition index (OI) was calculated as OI = (T-C)/(T + C), where T is the number of eggs on the treatment plate, and C is the number of eggs on the control plate. We also counted total eggs per cage across both the control and treatment to examine consistent female fecundity when generating the oviposition indices. However, in trials with the synthetic parasitoid odor mixture as our treatment, we note a significant decrease in the total observed fecundity or egg deposition, although all plant tissue experiments had nearly identical total egg numbers across the eight replicates (Fig. 5b).

### Trap Assays

As we had established a behavioral effect of these iridoids on egg laying, we next wanted to examine attraction and aversion using these odors. Experiments were performed in smaller plastic enclosures (10 cm × 10 cm × 8 cm), which contained two trap containers. To assist in total capture rates, we utilized red paper cones as the trap entrance. This has been shown to enhance capture for larger Drosophilids like *D. suzukii* (as opposed to smaller pipette tip entrances), as well as shown to enhance capture for those fly species that are perhaps more visually driven during search or host navigation paradigms (Keesey et al. 2019). One trap contained a damaged blueberry with 50 μl hexane solvent (control), while the other trap contained a damaged blueberry and 50 μl of treatment diluted in hexane. Both of the traps had 100 μl of laboratory-grade mineral oil added to assist in killing flies inside after they made a choice during the 24 hr experiments. Trials were conducted as noted before (12 hr Light:Dark cycle, 70% humidity, and 23 °C), with ten total replicates. The attraction index was calculated as AI = (T-C)/20 where a total of 20 flies were allowed to make a choice between the treatment (T) and control (C) trap containers.

### Statistical Analyses and Figure Generation

Statistical analyses were conducted using GraphPad InStat 3 (https://www.graphpad.com/scientific-software/instat/), while figures were organized and prepared using R Studio, Microsoft Excel and Adobe Illustrator CS5. Normally distributed data were analyzed using two-tailed, paired t-tests or a one-way analyses of variance (ANOVA; Tukey-Kramer multiple comparison test), where means with the same letter are not significantly different from one another. Boxplots represent the median (bold black line), quartiles (boxes), as well as the confidence intervals (whiskers). Color-filled boxplots denote significance from zero, while empty (white) boxes were not significantly different from zero. Error bars presented for bar graphs represent the standard deviation.

## Results

### Plant Chemistry

**Fig. 1:**
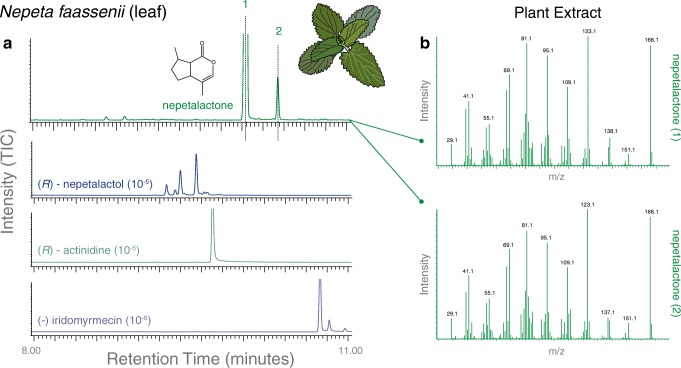
Gas chromatography mass spectrometry (GC-MS) analyses of *Nepeta* genus plants. **a** Shown at top is the leaf extract collected with hexane, and below are the three synthetic standards of the parasitoid pheromones for *Leptopilina boulardi* wasps, each odor of which has been shown to be aversive in several *Drosophila* species within the subgenus *Sophophora*, including *D. suzukii* adults and larvae (Ebrahim et al. 2015). There was no retention time or mass spectral overlap between *Nepeta* plant extracts and synthetic parasitoid compounds; however, *Nepeta* plants did contain high amounts of two unidentified isomers of nepetalactone. In total we tested 7 different species within this genus of plant, but did not find any matches for the parasitoid odors. **b** Mass spectra for the two main isomers of nepetalactone found in these plants

**Fig. 2:**
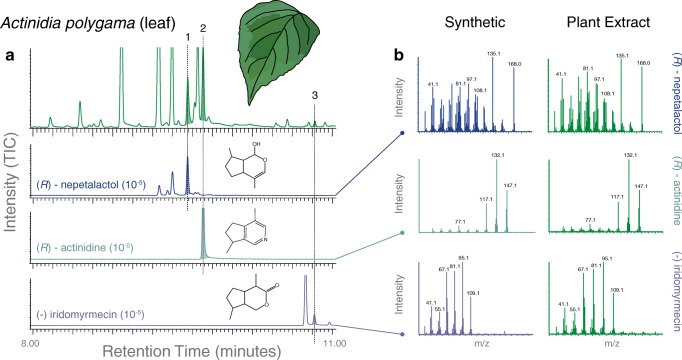
Gas chromatography mass spectrometry (GC-MS) analyses of *Actinidia* genus plants. **a** Shown at top is the leaf extract collected with hexane, and below are the three synthetic standards of the parasitoid pheromones for *Leptopilina boulardi* wasps, each odor of which has been shown to be aversive in several *Drosophila* species within the subgenus *Sophophora*, including *D. suzukii* adults and larvae (Ebrahim et al. 2015). In total we examined 4 species of *Actinidia*, including leaves and flowers from both male and female plants. Each tested sample contained potentially bioactive compounds, although those from *A. polygama* consistently contained the largest amounts. **b** Mass spectra for each of the three plant odors of interest and the three synthetic parasitoid compounds, where retention time and mass-spectral signature are nearly identical for all three odorants

**Fig. 3:**
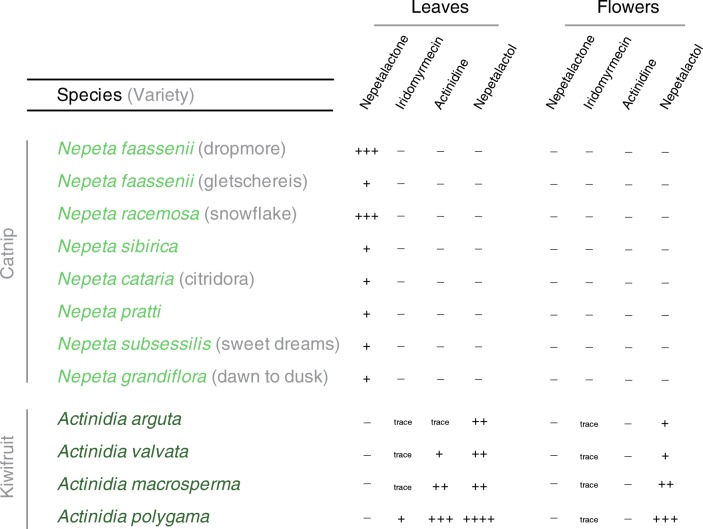
List of all tested plant extracts (leaves and flowers) for *Nepeta* and *Actinidia* species. Names of each species and variety that were tested, across both catnip (*Nepeta*) and kiwifruit (*Actinidia*) genera. While catnip varieties produced large amounts of unidentified isomers of nepetalactone, they did not generate any isomers of the behaviorally active compounds of interest. However, each species and genotype of kiwifruit produced one or several isomers of the parasitoid odors; moreover, leaf materials always contained larger amounts of the odors as compared to flowers. One species, *Actinidia polygama*, consistently produced the largest amount of our compounds of interest, and represents the best target for additional study of these iridoid odors, including the natural product that is the namesake of this genus of plant, actinidine. (− = odor was not detected by GC-MS; trace = threshold levels detected; + = low amounts detected; ++ = moderate amounts; ++++ = highest amounts of major iridoid components found)

We first sought to examine whether the plant extracts of any of the tested *Nepeta* or *Actinidia* species would indeed include the parasitoid compounds that are known to induce oviposition avoidance in Drosophilid flies (Ebrahim et al. 2015) (GC-MS; Fig. 1). For the seven *Nepeta* plant species, we identified large amounts of two as-of-yet unidentified isomers of nepetalactone in the leaves (but not in the flowers). However, we did not find any of the previously identified parasitoid odors (*i.e.* iridomyrmecin, nepetalactol, or actinidine) within these plant samples (Figs. 1, 3; Supplementary Figure 1). When we next examined the extracts of the four different *Actinidia* species, we found at least traces and in some species even large amounts of several iridoids that structurally resembled all three previously described parasitoid compounds (Figs. 2, 3). Here the consistently highest amounts of these three compounds were present in the leaf extracts of *A. polygama*. Also flower extracts of this species contained nepetalactol and some traces of iridomyrmecin (Fig. 3). We confirmed the presence, absence and identity of these plant-produced iridoid compounds by comparing the retention times as well as the mass spectral signature for each chromatogram peak to synthetic parasitoid reference standards (Figs. 1, 2a, b), where there was a close to perfect match for both retention time and mass spectral EI-MS data between the synthetic odorants and the plant-derived iridoid compounds from *Actinidia* species but not from any of the *Nepeta* varieties (Figs. 1, 2a, b).

### Electrophysiology of Parasitoid-Detecting Sensilla

**Fig. 4:**
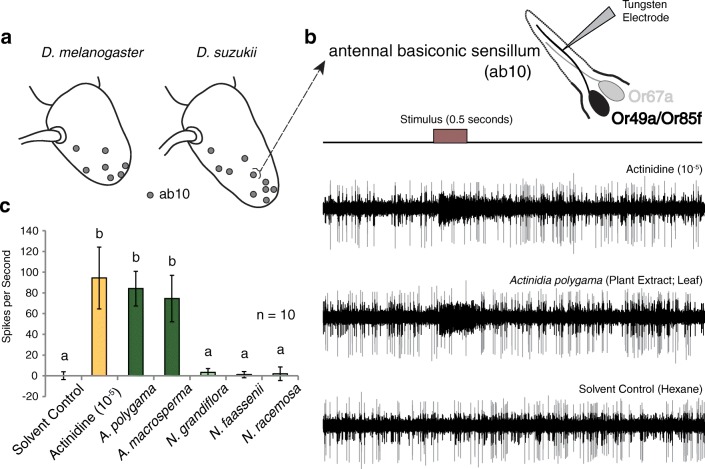
Single-sensillum recordings (SSR) from parasitoid-detecting olfactory receptors. **a** Schematic of *D. melanogaster* and *D. suzukii* (3rd antennal segment; funiculus) depicting locations of parasitoid-sensitive sensillum types. **b** Shown are single sensillum recordings (SSR) of antennal basiconic sensillum (ab10) responses towards the diagnostic parasitoid odor, actinidine, and towards the leaf extract of the plant species *Actinidia polygama*, as well as towards the solvent control, where each odor was tested across the same sensillum. Both the synthetic parasitoid odor and the extracted plant sample evoke similar responses in the same neuron, which has been shown to house Or49a and Or85f in *D. melanogaster* adults. **c** Quantified SSR responses towards plant extracts and the synthetic parasitoid odors, where only the tested *Actinidia* plant species produced activation of this neuron. These plant extracts were not significantly different in the strength of response from the synthetic odor (actinidine) in spikes per second, but the synthetic odor often produced a longer duration of activation than the plant samples. Means with the same letter are not significantly different from one another (α = 0.05)

Using a synthetic parasitoid pheromone compound, *Actinidine*, whose detection by the fly is highly specific and that activates only a single olfactory sensory neuron type (*i.e.* ab10B in *D. melanogaster*), we were able to map the locations in *D. melanogaster* as well as in *D. suzukii* of the antennal basiconic sensillum “ab10-like” types (Fig. 4a). This examination corresponded to and confirmed previous work on this receptor and sensillum type for *D. melanogaster* (Ebrahim et al. 2015). The overall body and antennal size of *D. suzukii* is significantly larger than *D. melanogaster*, as was shown recently (Keesey et al. 2019), thus it was not surprising that we noted more potential ab10 sensillum numbers for this pest species, simply because it has a higher total number of sensilla and more total basiconic sensilla than in *D. melanogaster* adults. We next tested whether any of the plant compounds would activate these same olfactory sensory neurons that are known to govern oviposition avoidance towards *Leptopilina* parasitoids (Fig. 4b). Here we tested the response of potential ab10 sensilla in *D. suzukii* towards both the diagnostic odor (actinidine, diluted to 10^−5^ in hexane) as well as hexane extracts from our *Actinidia* plant tissues (Fig. 4b). In the smaller “B” neuron of this sensillum type (which co-expresses Or49a and Or85f in *D. melanogaster*), we found strong responses to both the synthetic reference compound, actinidine, as well as to the leaf extracts of *A. polygama* and *A. macrosperma* (Fig. 4b, c). However, none of the extracts from *Nepeta* plants resulted in any activation of the neuron of interest (Fig. 4c), suggesting that the *Nepeta*-specific iridoids do not function as ligands for this neuron. The larger “A” neuron response profile for the ab10 sensillum of *D. suzukii* is also less sensitive in its response to benzyl butyrate, which is currently the best ligand for *D. melanogaster* flies. This neuron may thus be slightly different in its ligand spectra or odorant tuning compare to *D. melanogaster*. However, the “B” neuron showed nearly identical ligand spectra and odor sensitivity between the two tested *Drosophila* species. Here the synthetic parasitoid pheromones (*e.g.* actinidine) proved to be the best diagnostic odor for identifying ab10 sensillum types throughout the antenna of both fly species. Therefore, we conclude that the ab10B neuron appears to be highly conserved across the *Drosophila* genus, as has been suggested previously, and it is narrowly tuned specifically towards these iridoid compounds (Ebrahim et al. 2015).

### Behavioral Aversion with Plant Extracts and Parasitoid Odors

**Fig. 5:**
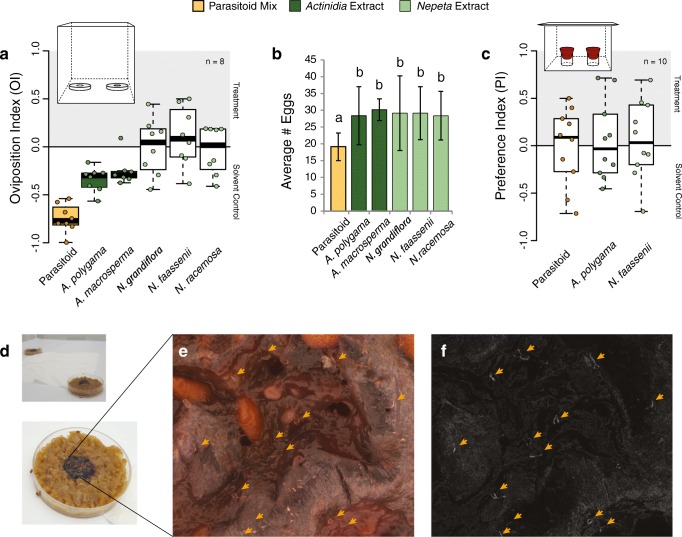
Ovipositional avoidance by *Drosophila suzukii* of parasitoid and *Actinidia* plant extracts. **a** Oviposition index for adult flies that were presented a choice between standard diet plates with solvent control or with treatment, where females preferred to lay more eggs on the side without parasitoid or *Actinidia* extracts. Only the two *Actinidia* plant extracts produced behavioral aversion while none of the three *Nepeta* species generated any significant preference. The mix of three synthetic parasitoid odors produced the strongest repellency for oviposition, or may have persisted longest in the environment. **b** Average number of eggs produced in each oviposition choice assay, where there was no significant difference in total eggs laid for any of the plant extracts, although the parasitoid mix again showed a significant reduction in total eggs deposited. **c** Attraction index (trap assays) of adult *Drosophila* towards the olfactory cues from the synthetic parasitoid mix or either genera of plant extracts as compared to the odor cues from the solvent control, where there is no significant attraction or avoidance shown to any treatment. **d** Oviposition assay paradigm, using two petri dish plates with damaged blueberries, which were separated by a white tissue paper that provided both a source of water and a standard distance of separation between control and treatment stimuli. **e** Color images were taken of oviposition sites in order to count egg numbers; however, due to the ability of *D. suzukii* females to completely bury their eggs deep within the substrate, it was often difficult to locate and count eggs. **f** Utilizing a black-and-white digital camera which was mounted to the same microscope, we could instead generate monochromatic or greyscale images, where it was far easier to discern eggs, even those completely buried in the substrate, via the identification of the white pairs of breathing tubes (highlighted with arrows). Means with the same letter are not significantly different from one another (α = 0.05). Color-filled boxplots denote significance from zero, while empty (white) boxes were not significantly different from zero. Error bars presented for bar graphs represent the standard deviation

Having shown that the *Actinidia* extracts contain parasitoid-like compounds via our GC-MS analyses, and subsequently, that these plant extracts can activate the fly olfactory circuit that is dedicated towards the avoidance of parasitoids, we next examined whether the plant extracts are sufficient to induce oviposition avoidance in *D. suzukii*. As shown previously (Ebrahim et al. 2015), when adult *D. melanogaster* flies were given the choice between oviposition plates with solvent control or plates with a mix of synthetic parasitoid pheromone odors, the adult females significantly preferred to lay eggs on the control, and avoided egg laying near the parasitoid odors (Fig. 5a). We found a similarly significant avoidance for oviposition when utilizing our new plant extracts taken from *A. polygama* and *A. macrosperma* (where *D. suzukii* females again preferred the solvent control over the treatment). However, no significant behavioral aversion was found for any of the three tested *Nepeta* plant extracts (Fig. 5a), even though these plants contained two isomers of nepetalactone, which are chemically similar in structure to the parasitoid odors. Thus, this neural circuit, both for detection via the antenna and for avoidance behavior, still appears to be narrowly tuned in all *Drosophila* species tested thus far. In addition to oviposition trials, we also examined the attraction index of adult *D. suzukii* towards the parasitoid odors and the plant extracts (Fig. 5c). Here, similar to the previously described behavior for *Drosophila* adults towards the body washes of *L. boulardi* (Ebrahim et al. 2015), we did not observe any significant behavioral aversion towards the parasitoid mix nor any of the plant extracts. This suggests that adult flies show only oviposition avoidance, and perhaps not any long-range repellency or feeding aversion towards these chemical stimuli. Future research will need to address either greenhouse or field-based testing of the efficacy of *Actinidia* plant extracts to combat *D. suzukii* oviposition.

## Discussion

The world-wide pest, *Drosophila suzukii*, oviposits in high value crops like cherries and soft fruits, including many kinds of berries and grapes. As this insect infests fruits just before harvest, any pesticide treatment is difficult to utilize due to the pesticide-specific pre-harvest intervals. We therefore investigated whether we could identify known oviposition deterrents for *D. suzukii* in natural, plant-based extracts that could in turn be customized towards novel IPM strategies while still presumably safe for human consumption. Like many other Drosophilid flies, *D. suzukii* detects and avoids the pheromone compounds of the parasitoid wasp *Leptopilina boulardi* (Ebrahim et al. 2015)*.* The highly specific olfactory circuit for this ovipositional avoidance has been described in detail for a close relative, *D. melanogaster*, where two olfactory receptors (OR49a and OR85f, which are co-expressed in one olfactory sensory neuron type, the ab10B olfactory sensory neuron (OSN) can detect the wasp-specific iridoid compounds, namely (−)-iridomyrmecin, (*R*)-actinidine, and (*S*)-nepetalactol. While activation of Or49a seems to be highly specific towards the parasitoid-specific isomer of iridomyrmecin, Or85f could be activated by several isomers of actinidine and nepetalactol in previous studies (Ebrahim et al. 2015). Both of these olfactory receptors (Or49a and Or85f) have also been described from the genome and from gene extractions of *D. suzukii* antennae (Ometto et al. 2013; Adrion et al. 2014; Ramasamy et al. 2016). Interestingly, *D. suzukii* also shows a potential gene duplication of Or49a (as compared to *D. melanogaster*, which only has a single copy), and this may in the future offer some behavioral significance in regard to studies of avoidance for other parasitoids or parasitoid odors (Ramasamy et al. 2016). For example, *D. suzukii* may show stronger aversion towards an as-of-yet-undescribed parasitoid species, or perhaps towards a native parasitoid (*i.e. Leptopilina japonica*), as provided by a novel odorant tuning via this receptor duplication. In *D. melanogaster* the activation of the ab10B neuron was demonstrated to be sufficient to govern oviposition avoidance that the flies exhibit in the presence of live parasitoid wasps (Ebrahim et al. 2015; Stökl et al. 2012). We therefore hypothesize that these similar compounds identified from *Actidinia* plants, which activated the same olfactory circuit, could potentially be applied in the field to repel *D. suzukii* flies from depositing eggs in fruit shortly before the harvest period.

Although we tested several plant species of the genera *Nepeta* and *Actinidia*, which are two genera that are known for their production of iridoid compounds, and while we identified a variety of iridoids from both plant genera, we found that only odorants from *Actinidia* were chemically similar (or potentially identical) to those previously identified from the parasitoid wasp, *L. boulardi* (Fig. 2). We furthermore found that only the *Actinidia* compounds and plant extracts were capable of activating the parasitoid-specific OSNs on the antennae of both *D. melanogaster* and *D. suzukii* flies during single-sensillum recordings (Fig. 4b). Moreover, that the iridoids from *Nepeta* plants were too chemically dissimilar from the original parasitoid compounds to serve as ligands for the wasp-specific olfactory receptors of the fly. Thus, not surprisingly, when we next tested behavior associated with plant extracts from both *Nepeta* and *Actinidia* plants in oviposition assays, we found avoidance only towards the *Actinidia* extracts, while the *Nepeta* extracts did not affect the oviposition choices of *D. suzukii* adults (Fig. 5a, b). It should be noted that we found stronger behavioral avoidance associated with the synthetic parasitoid odors than the *Actinidia* plant samples (Fig. 5a). This may be due to quantitative or qualitative differences between the plant extracts and the pure, isolated parasitoid odors, though future work is still needed to ascertain the rationale for differences that were observed. Similarly, additional work would be needed to examine any effect of these plant extracts on adult feeding or larval behavior.

Additional work is still needed to confirm the olfactory receptor (OR) identity of those receptors in *D. suzukii* adults that respond to these parasitoid and plant-based compounds. However, the identity of the olfactory receptors would not change the viability of plant extracts as a potential IPM technique towards reduction of oviposition in the field by this pest insect, especially given their functional similarity to those previously identified as key stimuli from the ab10 OSN type in *D. melanogaster* adults. That being said, the response profile and ligand spectra of the ab10B neuron, which expresses the olfactory receptors detecting both the plant and parasitoid odors (Fig. 4b, c), was functionally identical between the two examined *Drosophila* species in this study. Moreover, similarly responding olfactory sensory neurons (OSNs) were found in several other *Drosophila* species that were previously examined (Ebrahim et al. 2015), where all species that detected the wasp odorants also exhibited oviposition avoidance towards these identified parasitoid odors (Ebrahim et al. 2015). Thus we believe that the odorant receptors housed in the ab10B neuron most likely share a highly conserved amino acid structure across all species for this insect genus, similar to the high-rate of conservation of other aversion-related receptors such as Or56a (which detects geosmin (Stensmyr et al. 2012)). However, again, additional molecular genetic work is still needed to test this hypothesis across additional species. Correspondingly, we believe that the *Actinidia* extracts could act as potential oviposition deterrent for other Drosophilid species, but additional work is still needed to test other species beyond *D. suzukii* adults.

While field trials will be needed to test our proposed plant-based IPM strategy, for example with mixed cultivation using *A. polygama*, additional aspects also need to be addressed in tandem. For example, it is unclear whether these plant-based compounds from *Actinidia* plants have any behavioral effect on either parasitoid or predator recruitment (Glinwood et al. 1999; Zhang et al. 2006), which might provide additional benefits towards the biocontrol of *D. suzukii* in the field via increased predation or parasitism rates. Moreover, it is not known whether additional pest insects such as aphids, whose aggregation pheromone blend includes isomers of nepetalactone and nepetalactol, may also be increasingly attracted in the field by the compounds (Dawson et al. 1996), thereby creating additional agricultural risks or other direct harm to fruit production efforts. It is also unclear whether alley-cropping, agroforestry, or other push-pull strategies afforded by establishing *Actinidia* plants near, around or within fruit production can provide similar oviposition repellency to that observed in the laboratory. Alternatively, future research we need to address if only chemical extraction and application of *Actinidia* leaf extracts directly onto the crop will provide suitable quantities of avoidance cues for the efficacy of this IPM strategy to be successful. For example, dose response trials need to be conducted to ascertain effective application. The current conditions for growing *Actinidia* plants are similar to those for successful viticulture, thus vineyards may provide the most appropriate avenue for testing the planting of kiwifruit plants as a natural deterrent for *D. suzukii* across regions that already focus on wine production. However, given that *D. suzukii* is only an opportunistic pest of grapes, it may be more fitting to address alley cropping in berry fruit orchards instead. Future experiments in greenhouses will reveal whether the close vicinity of *Actinidia* plants is sufficient to reduce crop loss by oviposition of *D. suzukii* adults*.* Future analyses with additional plant species such as Indian nettle (*Acalypha indica*), valerian herbs (*Valeriana officinalisi*; *Nardostachys jatamansi*), snapdragon (*Catharanthus roseus*), yellowbells (*Tecoma stans*), and honeysuckle (*Lonicera caerulea*; *Lonicera tatarica*), which have all also been suggested to produce different kinds of iridoids (Bol et al. 2017); Scaffidi et al. 2016;, might reveal further potent *D. suzukii* or other Dipteran deterrents from natural or plant sources. It has also been shown previously that different isomers of iridoid compounds can have varying degrees of bioactivity (Civjan 2012; Ebrahim et al. 2015; Stökl et al. 2012), thus more work is needed to address stereochemistry of bioactive compounds. However, in *D. melanogaster*, this neural circuit has been shown to be very narrowly tuned (as are all known avoidance pathways in *Drosophila*), thus we feel it is unlikely to find many alternative iridoids.

While there is no perfect solution to the management of agricultural pests we currently encounter, perhaps due to rapidly evolving counter strategies by the insects or the microorganisms that we seek to combat, the scientific pursuits related to IPM as well as evolutionary neuroethology will continue to generate testable hypotheses and potential avenues for novel approaches to solve agricultural problems throughout the world. It is a growing concern that the wide-spread use of general pesticides such as neonicotinoids, which have been reported as harmful to bees and other pollinators, will only continue to hinder beneficial plant-insect interactions in natural environments (Van der Sluijs et al. 2013; Whitehorn et al. 2012). Thus again, we believe the possibility of utilizing natural-product chemistry, such as bioactive plant extracts or push-pull alley cropping, as opposed to the generation of new or more powerful pesticides, may afford a more sustainable and eco-friendly solution to pest insects such as *D. suzukii* for agriculture in the future.

## Electronic Supplementary Material

Supplementary Figure 1 **All varieties of*Nepeta*that were grown for this study.** Here we provide images for each species and each variety from this genus that we analyzed. In addition, we provide an overview of the chemistry from each plant type, where we highlight both isomers of nepetalactone (yellow and blue) in the figure. However, none of these plants produced any behavioral aversion. (PDF 23490 kb)

Supplementary Figure 2 **All synthetic isomers of parasitoid pheromones that were utilized.** (A-D) Each of the synthesized parasitoid odors are shown with their stereochemistry. More details for the production of these odors are available in the methods section. (PDF 286 kb)
