# Supplementary figures and images for: Plant-Based Natural Product Chemistry for Integrated Pest Management of Drosophila suzukii
Source: J Chem Ecol. 2019 Jul 1;45(7):626–37. doi: 10.1007/s10886-019-01085-1 (PMC6661260; doi:10.1007/s10886-019-01085-1)

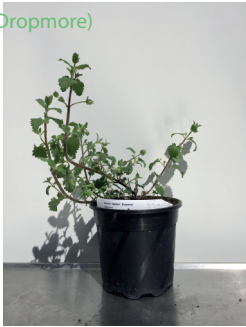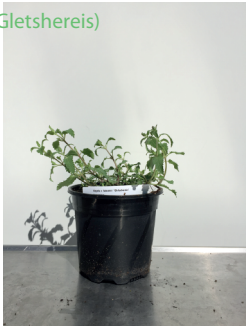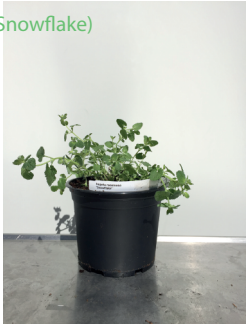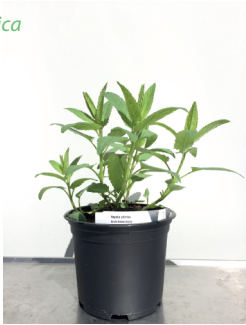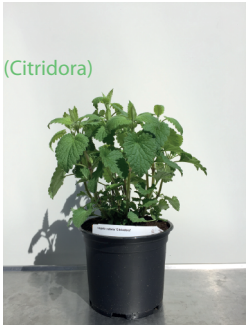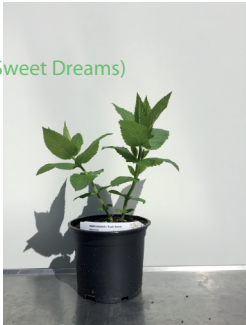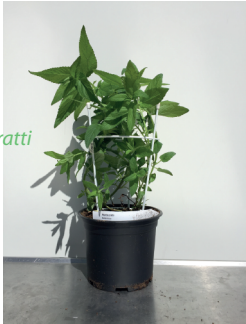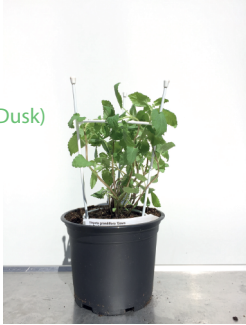

Supplement: Supplementary file 1 — All varieties ofNepetathat were grown for this study. Here we provide images for each species and each variety from this genus that we analyzed. In addition, we provide an overview of the chemistry from each plant type, where we highlight both isomers of nepetalactone (yellow and blue) in the figure. However, none of these plants produced any behavioral aversion. (PDF 23490 kb) [file 10886_2019_1085_MOESM1_ESM.pdf]
