# Supplementary material for: Plant-Based Natural Product Chemistry for Integrated Pest Management of Drosophila suzukii
Source: J Chem Ecol. 2019 Jul 1;45(7):626–37. doi: 10.1007/s10886-019-01085-1 (PMC6661260; doi:10.1007/s10886-019-01085-1)
Supplement: Supplementary file 2 — All synthetic isomers of parasitoid pheromones that were utilized. (A-D) Each of the synthesized parasitoid odors are shown with their stereochemistry. More details for the production of these odors are available in the methods section. (PDF 286 kb) [file 10886_2019_1085_MOESM2_ESM.pdf]

A

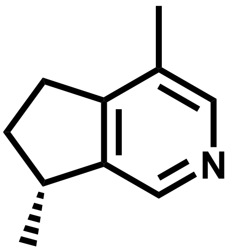

(*R*) - actinidine

B

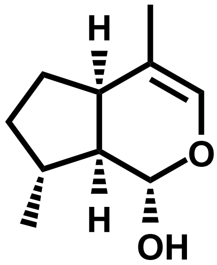

(1*S*, 4a*R*, 7*R*, 7a*S*) - nepetalactol
or
(-) - nepetalactol

C

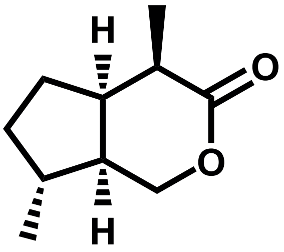

1:1

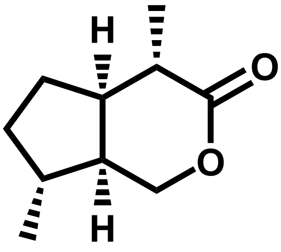

(4*R*, 4a*R*, 7*R*, 7a*S*) - iridomyrmecin
or
(-) - iridomyrmecin

(4*S*, 4a*R*, 7*R*, 7a*S*) - isoiridomyrmecin
or
(+) - isoiridomyrmecin

D

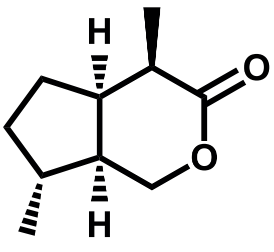

87% pure

(-) - iridomyrmecin
